# Topical Collection: International Year of Groundwater—managing future societal and environmental challenges

**DOI:** 10.1007/s10040-022-02587-1

**Published:** 2023-01-14

**Authors:** Marco Petitta, David Kreamer, Ian Davey, Jane Dottridge, Alan MacDonald, Viviana Re, Teodóra Szőcs

**Affiliations:** 1 Earth Sciences Department, Sapienza University of Rome, Roma, Italy; 2 Department of Geoscience, University of Nevada, Las Vegas, NV 89154-4010 USA; 3 International Association of Hydrogeologists, Reading, UK; 4 Mott MacDonald, Cambridge, UK; 5 British Geological Survey, Lyell Centre, Research Av South, Edinburgh, EH14 4AP UK; 6 Department of Earth Sciences, University of Pisa, Via S. Maria 53, 56126 Pisa, Italy; 7 Hydrogeology Department, Supervisory Authority for Regulatory Affairs, Budapest, Hungary

**Keywords:** Groundwater management, Decision making, Groundwater and society, Groundwater science communication, Sustainability

## Abstract

Groundwater’s role in maintaining the well-being of the planet is increasingly acknowledged. Only recently has society recognised groundwater as a key component of the water cycle. To improve public understanding and the proper use of groundwater, the hydrogeological community must expand its efforts in groundwater assessment, management, and communication. The International Association of Hydrogeologists (IAH) intends to help achieve the United Nation’s water-related Sustainable Development Goals (SDGs) by the adoption of innovative hydrogeological strategies. This essay introduces a topical collection that encapsulates IAH’s 2022 ‘Year for Groundwater’.

## Résumé

Le rôle des eaux souterraines dans le maintien du bien-être de la planète est de plus en plus reconnu. Ce n’est que récemment que la société a reconnu les eaux souterraines comme un élément clé du cycle de l’eau. Pour améliorer la compréhension du public et l’utilisation appropriée des eaux souterraines, la communauté hydrogéologique doit étendre ses efforts en matière d’évaluation, de gestion des eaux souterraines et de communication à leur sujet. L’Association Internationale des Hydrogéologues (AIH) entend contribuer à la réalisation des objectifs du développement durable (ODDs) des Nations unies liés à l’eau par l’adoption de stratégies hydrogéologiques innovantes. Cet essai présente une collection thématique qui résume l’‘Année pour les eaux souterraines’ 2022 de l’AIH.

## Resumen

El papel de las aguas subterráneas para mantener el bienestar del planeta está cada vez más reconocido. Sólo recientemente la sociedad ha reconocido que las aguas subterráneas son un componente clave del ciclo del agua. Para mejorar la comprensión pública y el uso adecuado de las aguas subterráneas, la comunidad hidrogeológica debe aumentar sus esfuerzos en materia de evaluación, gestión y comunicación de las aguas subterráneas. La Asociación Internacional de Hidrogeólogos (AIH) pretende contribuir a la concreción de los Objetivos de Desarrollo Sostenible (ODS) de las Naciones Unidas relacionados con el agua mediante la adopción de estrategias hidrogeológicas innovadoras. Este artículo presenta una colección temática que engloba el 2022 como ‘Año Internacional para las Aguas Subterráneas’ de la AIH.

## Abstract (zh)

地下水在维护地球福祉方面的作用日益得到认可。直到最近社会才深入认识到地下水是水循环的重要组成部分。为了提高公众对地下水的理解和合理利用，水文地质界必须扩大其在地下水评估、管理和交流方面的努力。国际水文地质学家协会(IAH)打算通过采用创新的水文地质学战略，帮助实现联合国与水有关的可持续发展目标(SDGs)。本文介绍了概括IAH的2022 “专为地下水的年”的专集。

## Riassunto

Il ruolo delle acque sotterranee nel mantenimento del benessere del pianeta è progressivamente riconosciuto, anche se soltanto recentemente la società ha accolto le acque sotterranee come componente chiave del ciclo dell’acqua. Per migliorare la comprensione del pubblico e l’uso ottimale delle risorse idriche sotterranee, la comunità idrogeologica deve ampliare i suoi sforzi nella valutazione, nella gestione e nella comunicazione delle acque sotterranee. L’Associazione Internazionale Idrogeologi (IAH) ha l’intenzione di supportare il raggiungimento degli obiettivi di sviluppo sostenibile (SDGs) delle Nazioni Unite relativi all’acqua, tramite l’adozione di strategie idrogeologiche innovative. Questo saggio introduce una collezione di articoli di attualità che sintetizza quanto realizzato dalla IAH nel 2022, anno dedicato alle acque sotterranee.

## Resumo

O papel das águas subterrâneas na manutenção do bem-estar do planeta é cada vez mais reconhecido. Só recentemente a sociedade reconheceu as águas subterrâneas como um componente chave do ciclo da água. Para melhorar a compreensão pública e o uso adequado das águas subterrâneas, a comunidade hidrogeológica deve expandir seus esforços na avaliação, gestão e comunicação das águas subterrâneas. A Associação Internacional de Hidrogeólogos (AIH) pretende ajudar a alcançar os Objetivos de Desenvolvimento Sustentável (ODS) das Nações Unidas relacionados à água por meio da adoção de estratégias hidrogeológicas inovadoras. Este ensaio apresenta uma coleção tópica que encapsula o ‘Ano para as Águas Subterrâneas’ de 2022 da AIH.

## Introduction

Hydrogeologists know that groundwater represents the hidden part of the water cycle and has a key role in human well-being, societal development, and environmental and planetary equilibrium (Re et al. 2022). This role is corroborated by the huge quantity (about 95% of planetary liquid freshwater) and high renewable rate of groundwater stored in the subsoil and aquifers (0.35 million km^3^ younger than 50 years, Gleeson et al. 2016). Hydrogeologists frequently neglect to disseminate this fundamental information both inside and outside the scientific community. At the same time, groundwater is still undervalued by governments and policymakers, who consider groundwater only in case of emergency/scarcity.

UN-Water and UNESCO dedicated the 2022 World Water Day to promoting the relevance of groundwater, “making the invisible visible”; thus, 2022 was considered a “year for groundwater” as stated in the World Water Development Report (United Nations 2022). Appropriately, the International Association of Hydrogeologists (IAH) has been playing a key role in several initiatives and promotes awareness of the significance of groundwater at different levels, from local to international. To this end, the IAH Commissions and Networks contributed to a topical collection published in this issue of the official journal of IAH, *Hydrogeology Journal.* Such contributions represent an update of the knowledge and achievements in groundwater science and demonstrate the passion that IAH members hold for communicating about groundwater. IAH provides representative global guidance on hydrogeology, looking both at the transfer of knowledge to external audiences and at evaluating the next challenges and potential solutions.

During the last decade, there has been a modification of the perception of environmental values and sustainable needs, which are summarised by the Sustainable Development Goals (SDGs; United Nations 2015). Because water plays a fundamental role in addressing all SDGs, and because groundwater is a major component of the hydrosphere, it should be included in many implementation processes previewed by the 2030 SDG agenda (specifically SDG6) with the goal to achieve universal and equitable access to safe and affordable drinking water for all. However, groundwater is often ignored in water and ecosystem management; thus, the benefits of groundwater—its availability, quality and accessibility—will not contribute to the successful achievement of the SDG goals, with negative impacts on society and the natural environment. To help incorporate groundwater more fully within the SDGs, several key actions are identified:

- Improvement in *communication* by hydrogeologists with other disciplines, policymakers and the public for “making the invisible visible” and to ensure that optimal policy and practice are based on an accurate and insightful understanding of groundwater systems
- Acceleration in the development of *new knowledge* within groundwater science, strongly interconnected with other disciplines, and driven by innovative approaches and techniques, to promote practical and sustainable solutions
- An engagement strategy, through *increased involvement of all stakeholders*, to guide the production of groundwater science that is useful, usable and accepted for guiding policy and practice (Milman and MacDonald 2020)
- To value and *integrate traditional knowledge* with the formal science of hydrogeology, thus to recognise the important role indigenous communities have in the management of groundwater resources

Hydrogeologists can contribute by expending more effort, adopting suitable tools, and facing expectations in the coming years. The application of this strategy will depend on differing economic, societal and geological conditions; technical solutions cannot be considered universal, but require flexible application depending on the local conditions, with different impacts and costs in different contexts.

## The main challenges

Several recent and new threats are impacting groundwater storage and quality, affecting services offered to society and to the natural environment. The impact of climate change on the water cycle is rapid and already visible, via the increased occurrence of extreme events (UNESCO, UN Water 2020) and alteration of aquifer recharge (IPCC 2022). The most frequent response to climate-change pressures occurs as an increase in groundwater withdrawals (Stigter et al. 2022; this issue). The visibility to the general public, particularly of extreme events such as droughts and floods, raises awareness of water issues, offering the possibility to underscore how groundwater is relevant to the adaptation of climate-change effects (Famiglietti 2014).

Human pressures are still affecting groundwater quality, causing pollution in highly urbanized systems, in agricultural and livestock areas, and also in less populated areas, where the limitations of remedial infrastructure and pollution prevention are a threat to groundwater resources (Misstear et al. 2022; this issue). Modern lifestyle has created a new generation of pollutants (emerging contaminants, such as pharmaceuticals, metabolites, personal care products, per- and polyfluoroalkyl substances (PFAS), micro- and nanoplastics), the occurrence of which are still underestimated due to a lack of analytical techniques and policy restrictions (Lapworth et al. 2022; this issue). At the same time, the spread of “classical” contaminants in groundwater has not stopped, due to food production requirements.

Ecological status and ecosystem services have been recognized as fundamental values, and they strongly depend on groundwater availability, defining the concept of groundwater-dependent ecosystems (Kreamer et al. 2015). Withdrawals and water-table decline can have a direct impact on ecological systems (De Graaf et al. 2019), which are quickly impacted before the water shortages can be understood by human users. This priority requires additional information resulting from the monitoring of aquifers even where no direct abstractions are active.

A widespread threat to groundwater quality is coastal marine intrusion (Stein et al. 2022; this issue), influenced by climate change (sea level rise) and increased groundwater withdrawals in coastal regions, resulting in water quality deterioration and ecosystem degradation.

The interaction of groundwater systems with surface waters (Boulton and Hancock 2006) is based on a fragile equilibrium, impacted both directly and indirectly by human pressures. The nexus between water, energy, food and ecosystems (WEFE nexus; UNESCO, EC, IWA 2021) is able to express the interdependence of these elements. Groundwater is a key element of the nexus, because its natural resilience can be considered as a self-regulatory contribution both in preserving the balance and smoothing any sudden changes.

Undoubtedly, a response to this incomplete list of challenges can be tackled by an increase in knowledge, additional surveys and data production, looking for solutions at different scales, from local cases to regional scale (Mádl-Szőnyi et al. 2022; this issue), up to synthesised worldwide information (BGR/UNESCO 2008; IGRAC 2021).

Addressing these challenges requires the direct involvement of society, at different stages and levels (Foster 2020), to promote actions for preserving groundwater quantity and quality by bottom-up and/or top-down approaches. Policy rules are an example of water governance; however, in many societies, practice is not dictated by policy, but rather by local norms. A socio-hydrogeology approach (Re 2021) is based on local community involvement, with a direct increase of awareness of water issues by the citizens, and with mutual learning through transdisciplinarity. At a wider scale, transboundary aquifers pose a management challenge that requires international cooperation to be effectively implemented (Rivera et al. 2022; this issue).

## The tools for possible solutions

How to face these and other challenges, not only to promote good management and protection of groundwater resources but also to achieve the more ambitious objectives of the SDGs, requires several robust tools. The classical hydrogeological tools (surveys, sampling, drilling, testing, analyses, conceptual models, mathematical models, etc.) remain the basic instruments for providing solutions for groundwater management. In addition, innovative tools are needed, or existing tools should be implemented in an innovative way. “Innovation” has been stated as a basic accelerator for SDGs achievement. Innovative approaches in groundwater science were discussed at the recent UN-Water Summit on Groundwater in Paris (France) in December 2022.

Innovations, to move beyond research into implementation, can be both technical and nontechnical. Successful innovations can be effectively translated into operational management and ultimately deliver governance outcomes. Innovation implies actions and priorities relevant to the local context. Although aquifers occur on regional to international scales, groundwater is developed and used at the local, sometimes even household, scale, in a more local and personal dimension. Optimization of sustainable groundwater use and ensuring continued good quality is, therefore, not only a question of applying the latest advances in technology, but also requires the active engagement of the user community.

General areas of innovation in groundwater resources include: (1) technological innovation and discoveries, (2) conceptual advancement such as improvements in problem-solving, policy actions, and regulatory controls, (3) upgrading educational approaches for students, professionals and young people, and (4) communication and social innovation.

The field of *technical innovation* is rapidly growing, benefiting international collaboration, interdisciplinary research, digital solutions, and the involvement of end-users. The latter is crucial to ensure the applicability of the technology to a variety of socio-economic and cultural backgrounds. Significant improvements have been observed in monitoring by using remote sensing techniques (Rodell et al. 2018), which have revolutionised the ability to identify decadal changes in groundwater storage.

The widespread adoption of pressure transducers linked to telemetry has given managers groundwater data at their fingertips, enabling near-real-time and online monitoring (Martinsen et al. 2022). Fibre-optic water temperature loggers have helped understand heat flow (Ruba et al. 2021), and the emerging technologies of quantum gravity sensors (Shearan et al. 2022) enable the monitoring of groundwater-storage changes at the field and catchment scale. At the single well scale, the Smart Handpumps initiative has reduced the time required for fixing a broken pump (Gamble et al. 2017), while solar-powered groundwater pumping (Closas and Rap 2017) rapidly gained popularity via a more affordable second generation of solar panels. Rapid tests for microbial contamination in drinking water can return instantaneous results for screening high-risk supplies (Sorensen et al. 2021). Managed aquifer recharge (Dillon et al. 2019) is also providing the ability to reuse appropriately treated urban stormwater, sewage and other waste waters to increase groundwater storage (Zheng et al. 2022; this issue).

In terms of *conceptual innovation*, water governance requires complex groundwater science to be simplified, while retaining the key elements, so that it can be understood by external actors. Regulatory agency actions and guidance, adopting innovative strategies for monitoring, control, and planning groundwater usage, give decision-makers the possibility to realize implementation strategies (European Commission 2008). Innovative inputs are frequently offered by agencies, academia and professional associations, producing new concepts and frameworks that can provide novel opportunities. Technical tools, such as mathematical models for groundwater flow and transport, as well as decision support tools, can be adopted by planners at city, regional and national scales. Policies and regulations posed by authorities can be an efficient way to regulate withdrawals, wastewater releases, water management and groundwater protection. Public consultations enhance the involvement of stakeholders in the policy process.

*Communication and social innovation* are essential to reduce the risk associated with any top-down action. Adopting a bottom-up process requires additional effort, putting people at the centre by involving local communities or even individual family members. The socio-hydrogeology network (Re 2021), operating at the interface between society and groundwater, has the goal of promoting the integration of social sciences into hydrogeology and including local perspectives by developing concepts on how inter- and transdisciplinary cooperation and research can be carried out.

Communication and dissemination actions include several tools: (1) the direct application of groundwater science to pressing challenges, as described in the IAH Strategic Overview Series (IAH 2022a); (2) the engagement of water end-users, well owners and civil society to help unveil the invisible connections between people and groundwater; (3) simple but effective tools represented by local radio programmes (e.g., IGRAC 2022), television documentaries, news media reporting and interviews with scientists and policymakers, which can be flanked by social media engagement and podcasts.

Finally, *educational innovation* will spread the relevance of groundwater to the wider public, particularly targeting the younger generation. Forced by the Covid-19 pandemic, widespread adoption of the Internet and online learning have enabled recent innovations in education. The availability of educational videos and archived webinars is growing every day, as are gaming approaches. The production of accessible, engaging, high-quality, educational materials, free of charge online in many languages, is supported by The Groundwater Project (Cherry et al. 2021).

Diversification of educational materials can be a powerful, yet simple innovation. The inclusion of nonwestern, traditional and indigenous knowledge contributes to an active engagement in decolonizing educational materials. This will provide a broader, more diverse, and more complete view of the knowledge spectrum that will result in the identification of efficient strategies for the long-term protection of groundwater better suited to individual local contexts.

Finally, IAH internal processes also require attention. The concerns and wider aspirations of IAH leading to 2030 are enshrined in the 2021 São Paulo-Brussels Groundwater Declaration (IAH 2022b). A strategy to improve the sustainability, equality and diversity of the organisation, by increasing membership, may include retaining members by providing benefits and good communication, education, outreach and knowledge transfer and collaboration with other organisations to promote understanding.

## Conclusions

Thanks to increasing international awareness of the key role of groundwater, not only in the water cycle, but also in intersectoral and interdisciplinary issues related to human and environmental needs, hydrogeologists now have the opportunity to offer significant contributions for a better future. The approach requires the adoption of a new strategy, founded on classical hydrogeological tools and accompanied by innovative tools, that has the dual aim of communicating the relevance of groundwater and accelerating successful implementation of the SDGs. One step forward may be this topical collection, “International Year of Groundwater”, which contains seven essays on relevant topics.

A wide range of innovative solutions and approaches is already available in both the technical and nontechnical areas, and there is an urgency with respect to their financing, adoption, and implementation. Application of these innovations and new cutting-edge practices are a cost-effective way.

The current challenges for the implementation of ground-breaking actions include: (1) promoting the widespread trial, adoption and review of novel and practical actions to maximise benefits, (2) ensuring that groundwater innovations are accessible and appropriate also for local conditions, and (3) enlisting agencies and governments to ensure a timely investment of resources to adequately support innovations and implement innovative actions.

All innovations need to be sustainable in the longer term and be accompanied/followed by excellent communication, education/training, community involvement, and maintenance. Innovations need the opportunity to be robustly tested in practice and evaluated to build evidence of sustainable success.

Here, a specific challenge is addressed to hydrogeologists: how to transform knowledge—based on field data, practical tools and conceptual models—into transferable results that can be easily understood by the different components of society, as well as by decision-makers, stakeholders and final users. The main additional efforts of hydrogeologists must be dedicated to improving communication, dissemination and educational skills, and simplifying the complexity of hydrogeologic groundwater systems and their interaction with other earth systems and human populations. It should be not so difficult, after making the invisible visible.
